# Optimized diffusion of buck semen for saving genetic variability in selected dairy goat populations

**DOI:** 10.1186/1471-2156-12-25

**Published:** 2011-02-09

**Authors:** Jean-Jacques Colleau, Virginie Clément, Pierre Martin, Isabelle Palhière

**Affiliations:** 1 INRA, UMR1313, Génétique Animale et Biologie Intégrative, 78352 Jouy-en-Josas, France; 2 Institut de l'Elevage, 31321 Castanet-Tolosan, France; 3 Capgenes, Agropole, 86550 Mignaloux-Beauvoir, France; 4 INRA, UR631 Station d'Amélioration Génétique des Animaux, 31326 Castanet-Tolosan, France

## Abstract

**Background:**

Current research on quantitative genetics has provided efficient guidelines for the sustainable management of selected populations: genetic gain is maximized while the loss of genetic diversity is maintained at a reasonable rate. However, actual selection schemes are complex, especially for large domestic species, and they have to take into account many operational constraints. This paper deals with the actual selection of dairy goats where the challenge is to optimize diffusion of buck semen on the field. Three objectives are considered simultaneously: i) natural service buck replacement (NSR); ii) goat replacement (GR); iii) semen distribution of young bucks to be progeny-tested. An appropriate optimization method is developed, which involves five analytical steps. Solutions are obtained by simulated annealing and the corresponding algorithms are presented in detail.

**Results:**

The whole procedure was tested on two French goat populations (Alpine and Saanen breeds) and the results presented in the abstract were based on the average of the two breeds. The procedure induced an immediate acceleration of genetic gain in comparison with the current annual genetic gain (0.15 genetic standard deviation unit), as shown by two facts. First, the genetic level of replacement natural service (NS) bucks was predicted, 1.5 years ahead at the moment of reproduction, to be equivalent to that of the progeny-tested bucks in service, born from the current breeding scheme. Second, the genetic level of replacement goats was much higher than that of their dams (0.86 unit), which represented 6 years of selection, although dams were only 3 years older than their replacement daughters. This improved genetic gain could be achieved while decreasing inbreeding coefficients substantially. Inbreeding coefficients (%) of NS bucks was lower than that of the progeny-tested bucks (-0.17). Goats were also less inbred than their dams (-0.67).

**Conclusions:**

It was possible to account for complex operational constraints while developing goat selection schemes, both efficient and sustainable. Therefore, the recommended selection and mating decisions might receive attention from goat breeders using both AI and NS.

## Background

It is increasingly accepted that selection procedures should be as sustainable as possible, both to avoid a fast loss of variability in the genetic pool of selected populations and to avoid inbreeding effects on performances. The principles of these procedures have become quite clear, based on extensive research and validation work [1,2]. The major challenge will be to implement these procedures in the field. For any selection scheme, the appropriate approach is to first identify the detailed selection steps and corresponding operational constraints, then to accordingly develop optimization algorithms. This approach is much more demanding for large domestic animals than for poultry or fish for instance, due to many existing factors such as overlapping generations, longer lifetime, a variety of reproduction methods, and a large number of breeding farms [3,4]. Furthermore, in these species, breeders are mainly interested in obtaining genetic gains for some important traits. Therefore, the appropriate approach should also provide a reasonable trade-off between breeders' demands and maintenance of genetic variability. An example of selection or mating recommendations acceptable to breeders was given in [5].

Goat breeding schemes developed later than their dairy cattle or pig counterparts, mainly because of the slow development of artificial insemination (AI) over years. Collecting semen from bucks is much more difficult than from bulls. Furthermore, natural service (NS) bucks should be used on the females not eligible for AI (due to bad reproduction profile) and also on every female returning to heat because goats are inseminated only once. Since 2002, French goat breeders have paid attention to the management of genetic variability and, consequently, they might be interested in following the guidelines mentioned above. From 2002 to 2005, AI bucks were selected within families (12 grand-sire families) and, from 2006 onwards, young males to be progeny-tested were produced in a two-step procedure optimizing contributions and then mating scheme. The mating scheme proposed during this period was widely accepted in practice.

The objective of this study was to present a comprehensive optimization procedure for completing the management of genetic variability in these selected goat populations. The main goal of the procedure is to optimize the contributions of progeny-tested bucks, used for replacement of females or NS bucks, and to propose appropriate matings. The current situation is not satisfactory because no recommendation has been given to breeders for these purposes, especially on the choice of AI sires to produce future young NS bucks (a usual practice). An additional goal was to optimize the distribution over flocks of semen from young bucks to be progeny-tested, in order to improve the quality of breeding value evaluation. Finally, the procedure was tested on a real data set involving the two main breeds of dairy goats used in France, *i.e.*, the Saanen and the Alpine breeds.

## Methods

### General outline

The multiplicity of objectives led to a five-step approach. In each step or sub-step, the optimization algorithm aimed at maximizing the value of a locally-defined function that often accounted for several components. Simulated annealing [6-9] was used throughout, due to its power and its flexibility. When constraints were to be fulfilled, *e.g.*, to increase acceptability by breeders, an adaptive simulated annealing was used, following the approach described in [5]. For better acceptability, breeders were allowed to define their private selection objective. In the data set analyzed, three possible breeding objectives were used in all steps except for step 3: the "dairy" breeding objective (ICC), available since 2000 [10], the "udder shape" breeding objective (IMC) and the overall breeding objective combining both (IG), available since 2006 [11]. In step 3, a single breeding objective had to be considered and the overall breeding objective (IG) was chosen. For the simulated annealing procedures to be executed in unbiased conditions, the corresponding EBVs were expressed in genetic standard deviation units. The five-step approached involved:

### Step 1: replacement of NS bucks (NSR)

First, the number *n *of future replacement males was calculated in each flock based on its size N, considering all the breeding females (inseminated or not). A single NS buck was considered to be unable to serve more than 50 goats. Then, *n *= ceiling (N/50). For instance, if N = 120, then *n *= 3. Understandably, these *n *young bucks should be sons of *n *different AI progeny-tested bucks. This step also included selection of buck dams (4 per AI sire). In this case, 12 buck dams should be used. To maximize acceptability by breeders, the buck dams were the best females for the selection objective chosen in their flocks. The aim of this step was to optimize the choice of these *n *different AI sires in each flock, given the pre-selected buck dams.

The AI progeny-tested sires to be used on buck dams were chosen after considering the constraints mentioned above and the amount of available doses The local function to be used in each flock involved the estimated breeding value (EBV) of each candidate for the private breeding objective and his average coancestry with the flock females (inseminated or not), to prevent an excessive development of inbreeding through NS.

Two penalties (positive or null values) were defined for each progeny-tested buck *k *used in flock *i*. The trait penalty *π*_*T*,*ik *_was equal to *Max*(*T*_*ik*_) - *T*_*ik *_where the *T*_*i*_'s referred to the standardized EBV's for the selection objective of flock *i*. The coancestry penalty *π*_*P,ij *_was the maximum coancestry between buck *k *and a buck dam from flock *i*. The average penalties for flock *i *were ${\bar{\pi }}_{T,i}$ and ${\bar{\pi }}_{P,i}$. For the whole NS population, the average population penalties were ${\bar{\pi }}_{T}$ and ${\bar{\pi }}_{P}$. Therefore $E\left({\bar{\pi }}_{T}\right)$ and $E\left({\bar{\pi }}_{P}\right)$ were the expected population penalties under a random choice of AI bucks.

These expectations were calculated numerically, based on 200 replicates, because this choice depended on several constraints (doses available, strictly 4 mates per combination flock*buck). After running two distinct simulated annealings, the minimal populations penalties were $Min({\bar{\pi }}_{T})$ and $Min({\bar{\pi }}_{P})$. Finally, the performances of a given solution were evaluated by 2 ratios ranging from 0 (random matings) to 1 (specialized annealing) denoted hereafter 'penalty reduction'.

$$\rho_{T}=\frac{E\left({\bar{\pi }}_{T}\right)-{\bar{\pi }}_{T}}{E\left({\bar{\pi }}_{T}\right)-Min\left({\bar{\pi }}_{T}\right)}\;\text{and}\;\rho_{P}=\frac{E\left({\bar{\pi }}_{P}\right)-{\bar{\pi }}_{P}}{E\left({\bar{\pi }}_{P}\right)-Min\left({\bar{\pi }}_{P}\right)}$$

The main function *f *to be maximized by simulated annealing was $f=\frac{\rho_{T}+\rho_{P}}{2}$. Furthermore, the constraint function *f*_1 _= (*ρ_z _*- *ρ_P_*)^2 ^was defined in order to provide a completely balanced efficiency for both components.

The alternative solutions tested in the simulated annealing were provided by permutations. For instance, in flock *i*, chosen randomly, the 4 semen doses of buck *k *used in flock *i *were permuted with 4 unused doses of buck *l *still not considered in this flock. Complications arose, especially for small flocks, where the subsequent optimization might nevertheless lead to a high maximum inbreeding coefficient or to a high maximum number of faults (a mating was considered faulty for a trait when the corresponding EBV belonged to the worst 20% of all the possible matings, across flocks, between all the females to be inseminated and all the progeny-tested sires available). Then, during the simulated annealing, the buck *l *mentioned previously was chosen in order to exhibit a maximum coancestry coefficient with all the flock buck dams lower than a limit and/or to generate matings with less than 3 faults. The limit of coancestry was 5% when a single buck had to be chosen and 10% otherwise.

After choosing the sires of the future NS bucks, matings with buck dams were finally optimized both for inbreeding and the number of faults for the three progeny's EBVs (ICC, IMC and IG). Optimisation was conducted separately for each flock involving at least 2 bucks.

In flock *i*, the average penalties were ${\bar{\pi }}_{iF}$ and ${\bar{\pi }}_{iD}$. The penalty reductions were$\rho_{iF}=\frac{E\left({\bar{\pi }}_{iF}\right)-{\bar{\pi }}_{iF}}{E\left({\bar{\pi }}_{iF}\right)-Min\left({\bar{\pi }}_{iF}\right)}$ and $\rho_{iD}=\frac{E\left({\bar{\pi }}_{iD}\right)-{\bar{\pi }}_{iD}}{E\left({\bar{\pi }}_{iD}\right)-Min\left({\bar{\pi }}_{iD}\right)}$. The main function *f *to be maximized by simulated annealing was $f=\frac{\rho_{iF}+\rho_{iD}}{2}$ and the constraint function was *f*_1 _= (*ρ_iF _*- *ρ_iD_*)^2^.

### Step 2: allocation of young AI bucks to flocks for progeny-testing

This step determined the number of mates per combinations flock*young buck, without any reference to the potential breeding value of these young bucks or to potential inbreeding. The procedure searched for a maximal dispersion of bucks over flocks, ensuring a high connectedness between herds for young bucks' evaluations, under the constraint that the proportion of inseminations by young bucks was constant in any flock (30%). First, the number of mates allocated to each young buck minimized the variance of individual allocations, based on the following procedure.

Let *m_k _*be the number of mates to be found for young buck *k*, given that the overall sum $M\;=\;\sum_{k}m_{k}$ is already known. Then, *i) *set *m_k _*= *Min *(*d_k_*) for any *k *, where *d_k _*is the number of doses for buck *k; ii) *increase the *m' *s by 1 when possible (comparison with the *d*'s) and flag the bucks where *m *= *d; iii*) if $S\;=\;\sum_{k}m_{k}\;<\;M$, then go to *ii*); if S = M, then stop; if S = M+*x*, then decrement by 1 the *m' *s of *x *unflagged bucks, chosen randomly, and stop.

Given the overall sire allocations and given the number of mates devoted to progeny-testing in each flock, the detailed allocations per combination sire*flock were determined by simulated annealing, after minimizing the sum over flocks of squared within flock buck frequencies. This procedure led to a maximum dispersion of young bucks within flock.

### Step 3: contribution of progeny-tested bucks to female replacement

This step determined the overall numbers of mates allocated to the selected progeny-tested bucks, accounting for the results of step 1. The objective was to maximize the average overall EBV of the selected bucks, while minimizing the average difference Max(EBV) - EBV, and to minimize the average coancestry coefficient in the whole female population, augmented with the future females to be born (0.45 expected female progeny per goat inseminated), after considering the young females to be born from the AI planned for NSR and progeny-testing.

The optimization was carried out to maximize an average penalty reduction *ρ *through simulated annealing (permutations between unused and used doses). The bounds needed for computing such a reduction were provided *i) *by the average penalties incurred by choosing bucks randomly from the semen stores not used in step 1; *ii) *by the minimal penalties obtained after running specialized simulated annealings.

In fact, the average coancestry in the augmented population could be decomposed into a constant term and a variable term depending on the contributions found. We only considered the variable term, *i.e. *we used a 'partial' function. Let **x **be the vector of buck frequencies and **Α **the relationship matrix between bucks. Vector **x **could be decomposed into $\left(\begin{array}{l} x_{0}\\ x_{1}+s_{1} \end{array}\right)$ where the first element pertained to young bucks and progeny-tested bucks with stores of semen doses exhausted after step 1 (bucks called "0"), the second element pertained to the other bucks (bucks called "1"), already used with frequency vector **s**_1 _in step 1. Then, after rescaling, the function to minimize was $\frac{1}{2}x_{1}^{'}A_{11}x_{1}+\omega^{'}x_{1}$ where $\omega =\frac{1}{2}A_{11}s_{1}+A_{10}x_{0}+\frac{1}{2}\alpha a$, **a **was the vector of the average relationships between bucks "1" and inseminated females, and *α *was a constant depending on *p*, the expected number of female progeny per inseminated dam (*α *= 1 + 2/*p*). In the data set, *p *was equal to 0.45.

### Step 4: mating design on regular females for female replacement

Here, young and progeny-tested bucks were considered at the same time, taking into account the results of steps 1 to 3. The three purposes were: first, to minimize the inbreeding coefficient, second, to maximize local adequacy of the progeny-tested bucks in each flock and third, to maximize the number of different bucks in each flock, in that order. A particular mating was referenced by the quadruplet *ijkl*, where *i *was the flock, *j *was the buck category (tested or young), *k *was the buck within category *j *and *l *was the female within flock *i*. This mating incurred an inbreeding penalty *π*_*F*,*ijkl *_that was equal to the coancestry coefficient between buck *jk *and female *il*, plus a trait penalty for tested bucks, *π*_*T*,*ikl *_= *Max*_*k *_(*T*_*ik*_) - *T*_*ik*_.

For flock *i*, after combining buck categories, the average penalties were ${\bar{\pi }}_{F,i}$ and ${\bar{\pi }}_{T,i}$. The average population penalties were ${\bar{\pi }}_{F}$ and ${\bar{\pi }}_{T}$. Finally, penalty reduction ratios were $\rho_{F}=\frac{E\left({\bar{\pi }}_{F}\right)-{\bar{\pi }}_{F}}{E\left({\bar{\pi }}_{F}\right)-Min\left({\bar{\pi }}_{F}\right)}$ and $\rho_{T}=\frac{E\left({\bar{\pi }}_{T}\right)-{\bar{\pi }}_{T}}{E\left({\bar{\pi }}_{T}\right)-Min\left({\bar{\pi }}_{T}\right)}$. The main function to be maximized was $f=\frac{\rho_{F}+\rho_{T}}{2}$ and the first constraint function was *f*_1 _= (*ρ_F _*- *ρ_T_*)^2^. Moreover, the third penalty was the average within-flock variance of sire frequencies ${\bar{\pi }}_{c}$ and the second constraint function was $f_{2}={\left({\bar{\pi }}_{c}-{\tilde{\pi }}_{c}\right)}^{2}$. The desired value, ${\tilde{\pi }}_{c}$, was determined so that only 10% of the flocks exhibited sire concentration issues. Such an issue occurred when r at least one AI sire used both for male or female replacement or at least one progeny-tested sire allocation higher than 10% of the females inseminated by progeny-tested sires. Alternative solutions were obtained from two possible processes depending on the randomly sampled female. If mated to a young buck, then process 1: sample randomly within flock a female mated to a progeny-tested buck and then permute bucks. If mated to a progeny-tested buck, then process 2: sample randomly in another flock a female mated to a progeny-tested buck and then permute bucks. Process 1 modified *ρ_F _*whereas process 2 modified both *ρ_F _*and *ρ_T_*.

### Step 5: NS value of males from regular matings

The probability of obtaining no male progeny from a given AI sire (mated to 4 buck dams) in step 1, was 0.22 and was the result of the following parameters: prolificacy (1.5), sex-ratio (0.50), AI fertility (0.60), and expected rejection rate by breeders, based on phenotypes (0.20). Consequently, step 5 was introduced for detecting regular matings acceptable for NS replacement.

A linear function was computed in each flock to discriminate the 'official' matings for NS replacement from the matings between regular females and progeny-tested bucks. The values of the latter matings were expressed based on this function. In a given flock, all the matings between all the females to be inseminated and all the progeny-tested available bucks were considered. Matings 1 were the matings selected for NS replacement and matings 2 were the others. Each mating was described by two variables, attached to the corresponding progeny. Variable 1 was the EBV (= half parental EBV) for the flock selection objective and variable 2 was the average coancestry with all the females of the flock. These variables were standardized, leading to variables *x*_1 _and *x*_2_. On the new scale, the average differences between matings 1 and 2 for these variables were *d*_1 _and *d*_2_. The discriminant function maximizing the ratio $\frac{{\left(d_{1}+\omega d_{2}\right)}^{2}}{\mathrm{var}\left(D\right)}$ was *D*(*x*_1_, *x*_2_) = *x*_1 _+ *ωx*_2_, where $\omega =\frac{d_{2}-rd_{1}}{d_{1}-rd_{2}}$ and where *r *was the correlation coefficient between variates. For the sake of simplicity, results were given in integer scores: 0 for the matings with young bucks, 10 for the official matings and 1 to 9 for the other matings. The worst mating for the D function was scored 1 and the matings better than at least one official mating for the D function were scored 9. Generally, the practical range of NS values was 3-8 and matings scored 7-8 might be considered in practice for replacing missing males in step 1.

### Data set

The test data were provided by Capgenes, the organization in charge of the breeding scheme, and was on 65,734 goats (38,543 Alpine and 27,191 Saanen) and 156 AI bucks (88 Alpine and 68 Saanen). Goats actually inseminated in 2006 using semen from both progeny-tested sires and young bucks were considered. Consequently, they mostly belonged to the selection flocks (85%). Elite dams producing young AI bucks (about 600 Alpine and 400 Saanen) were not included in the data set because this step was treated separately by another optimization procedure (see Introduction). The number of different flocks involved was 1205 (697 Alpine flocks and 508 Saanen flocks). The flocks using both breeds were considered as two different flocks because the optimization procedures were carried out separately within breed. The number of females inseminated per flock was, on average, 55 in Alpine flocks (standard deviation = 50) and 54 in Saanen flocks (standard deviation = 52), and represented about 27% of the total number of females in the flock. This figure departed substantially from the average % of AI on the whole population the same year (9%) due to the involvement of test flocks in selection schemes.

The males included in the test data corresponded to AI progeny tested bucks (45 in Alpine and 39 Saanen) and young bucks to be progeny tested (43 Alpine and 29 Saanen). The usual objective of the breeding organization has been to serve at least 200 females per young buck and 30% of females for each flock by young bucks. The average number of doses available from young male was 320 (standard deviation = 41) for Alpine and 356 (standard deviation = 57) for Saanen. This represented 119% of the needs for progeny-testing for Alpine and 127% for Saanen. As to AI proven sires, all the candidates for service existing in 2006 were selected. The number of doses by buck ranged from 150 to 2,467 with an average of 1,085 for Alpine and 1,040 for Saanen. Considering that 70% of females would be mated with semen from these bucks, the supply corresponded to 181% of the needs for Alpine and 213% for Saanen.

Breeders were asked about the general selection objective for their flock (*i.e.*, the traits they wanted to focus on). Among the 263 responding breeders, 126 chose ICC, 34 IMC and 103 IG. Flocks without declared objective were merged into the IG group and 87% of the flocks belonged to this group. Due to this heterogeneity of objectives, the EBVs corresponding to each local objective were called 'local merit' and expressed in local *σ_g_*.

Table 1 shows the population statistics of progeny-tested sires and females (candidates for selection) for local merit, inbreeding and coancestry with flock. Sires were weighted according to their available semen doses. Coancestry of males meant coancestry with all the females and coancestry of females meant coancestry with the females of the same flock.

**Table 1:** Average local merit, average inbreeding and coancestry of candidates (parents), according to breed and sex

Breed	Sex	Merit (local *σ_g_*)	F (%)	Coancestry* (%)
Alpine	Tested bucks	2.16	2.28	2.29

Alpine	Females	1.00	1.75	3.61

Saanen	Tested bucks	2.08	2.25	2.72

Saanen	Females	1.01	1.98	3.84

*For tested bucks: coancestry with all the females. For females: coancestry with females of the same flock

Inbreeding and coancestry coefficients were calculated according to the indirect method [12,13]. Pedigrees of males were fairly complete but completeness of female pedigrees varied substantially (Table 2). An important part of this variation was between flocks (56% and 38%, respectively) due to differences in AI rate and sire recording after natural service.

**Table 2:** Age (in years) and number of effective generations known (in years) in pedigrees per breed and category of candidates

Breed	Category	Age Average	**Age St. dev**.	No of effective generations Average	**No of effective generations St. dev**.
Alpine	Tested bucks	5.7	1.1	7.6	0.4

Alpine	Young bucks	1.6	0.1	8.5	0.4

Alpine	Dams	3.0	1.3	6.7	2.8

Saanen	Tested bucks	5.2	1.5	6.9	0.5

Saanen	Young bucks	1.6	0.1	8.0	0.4

Saanen	Dams	2.9	1.3	6.5	2.9

## Results

Table 3 for the Alpine breed and Table 4 for the Saanen breed show the characteristics of the future progeny and corresponding parents for the three types of matings for Alpine and Saanen breeds, respectively. The three types of mating included those to produce young NS males, female replacement and young sires for progeny testing.

**Table 3:** Overall results for the Alpine breed: buck replacement (NSR), goat replacement (GR), progeny-testing (PT)

Animals involved	Merit (local *σ_g_*)	F (%)	Coancestry* (%)
Progeny NSR	2.53	1.75	2.96

Sire NSR	2.94	2.30	2.00

Dam NSR	2.12	1.91	3.90

Progeny GR	1.51	1.12	2.81

Sire GR	2.32	1.78	2.00

Dam GR	0.70	1.87	3.61

Progeny PT		1.29	2.79

Sire PT		2.33	2.16

Dam PT	0.71	1.50	3.43

*For sire: coancestry with all the females. For dams and progeny: coancestry with females of the same flock.

** NS value is the score of male progeny for replacing NS bucks

**Table 4:** Overall results for the Saanen breed: buck replacement (NSR), goat replacement (GR), progeny-testing (PT)

Animals involved	Merit (local *σ_g_*)	F (%)	Coancestry* (%)
Progeny NSR	2.34	1.97	3.21

Sire NSR	2.67	1.76	2.30

Dam NSR	2.00	2.18	4.10

Progeny GR	1.58	1.29	3.08

Sire GR	2.51	1.73	2.31

Dam GR	0.66	2.18	3.85

Progeny PT		1.42	3.06

Sire PT		2.52	2.45

Dam PT	0.64	1.60	3.67

*For sire: coancestry with all the females. For dams and progeny: coancestry with females of the same flock.

** NS value is the score of male progeny for replacing NS bucks

The NSR phase was very efficient for increasing the local merit of male progeny. The efficiency parameter *ρ *(see Methods step 1) was equal to 83% and 81% for the Alpine and Saanen breeds, respectively. Such a high efficiency was due to the power of simulated annealing and to the fact that this phase was given the priority of access to semen doses. The NS male progeny should be compared to contemporary progeny-tested sires, when they will be used for reproduction in flocks, i.e. at about 1.5 year. Given that the annual genetic gain for the overall selection objective is about 0.15 genetic standard deviation, the NS male progeny will compete with AI sires better by 0.22 (= 0.15 × 1.5) than the current sires of Table 1. The corresponding comparison shows that the NS male progeny will not be inferior to their AI competitors for merit. However, despite the procedure, they will be more related to their flocks than the AI sires (see Table 1), due to the coancestry between dams and other females of their flocks.

The local merit of female progeny was much higher than for their dams and corresponded to about 5 and 6 years of annual genetic gains for the Alpine and the Saanen breed respectively. This gain was much higher than expected from the age of dams (about 3 years, see Table 2). Consequently, the proposed mating scheme was expected to increase the future annual genetic gain. It also resulted in making female progeny being less related with flock females than their dams. It can be noted that these favorable results were obtained despite the fact that the corresponding procedure considered semen stores partly exhausted by step 1. The efficiency parameter ρ was equal to 54% and 64% for the Alpine and Saanen breeds, respectively. Inbreeding of progeny was also substantially lower than inbreeding of dams.

The procedure intending to equalize distribution of semen between young sires to be progeny-tested was efficient as shown by the standard deviation of the number of mates per young buck which was 33.5 and 35.7 for the Alpine and the Saanen breeds respectively (to be compared to 41 and 57 for the number of available doses). Dissemination of semen across flocks was also efficient. Most of the time (91% and 80% in Alpine and Saanen breeds, respectively), young sires were used on a single mate in each flock.

The frequency of cells with more than three mates was 0% in Alpine breed and 9% in Saanen breed. Cells with at least 2 mates could not be avoided in large flocks where the number of females devoted to progeny-testing was larger than the number of young sires. Step 1 used the very best females in each flock, as explained previously. Table 3 and 4 clearly show that on the other females, their allocation to young sire or to tested sires was quite independent of their genetic merit. In both breeds, matings with young sires generated slightly more inbreeding than mating with tested sires. This might be due to the fact that young sires' pedigrees were a little bit longer.

Though planned in step 1, obtaining males from an AI sire mated to a batch of 4 in a given flock was uncertain, as mentioned previously. The failure probability was 22% per AI sire if 4 dams could be programmed. Therefore, the potential of males born from the matings first dedicated to replacement of females had to be examined. Table 5 shows the frequency, average local genetic merit and average coancestry of NS male progeny with females of the flock, for different classes of NS values. Based on this table, scores 7-9 would be acceptable. In comparison with score 10, merits were lower but coancestries with females of the flock were reduced. Other scores were unfavorable for merits and coancestries. Monte-Carlo simulation with 500 replicates was performed in order to assess the score probabilities for the males ultimately chosen for NS replacement. Table 6 shows the results of the simulation. Based on this table, a score of at least 7 was obtained in 95.3% and 96.9% of cases for the Alpine and the Saanen breeds, respectively. As a consequence, the average expected merit of chosen males for NS was 2.26 and 2.08, respectively compared to 2.53 and 2.34 in Table 5 (a drop of about 0.27). The merit of future males was predicted to be similar to that of contemporary AI sires.

**Table 5:** Frequency, average local merit, average coancestry of NS male progeny with females of the flock, for different classes of NS values

Breed	Alpine	Alpine	Alpine	Saanen	Saanen	Saanen
**NS value**	**Freq. (%)**	**Merit (local σ*_g _*)**	**Coancestry (%)**	**Freq. (%)**	**Merit (local σ*_g _*)**	**Coancestry (%)**

10	21.3	2.53	2.95	21.4	2.34	3.17

9	9.9	1.93	2.61	15.7	1.95	2.84

8	8.4	1.66	2.60	9.6	1.63	2.92

7	10.0	1.50	2.74	9.3	1.43	3.13

6	8.8	1.34	1.93	6.0	1.23	3.40

5	5.0	1.13	3.16	3.0	0.98	3.62

4	2.2	0.90	3.30	0.9	0.75	3.94

3	0.5	0.62	3.57	0.1	0.41	3.80

**Table 6:** Frequency of NS values of young males ultimately chosen for NS (simulation with 500 replicates)

NS value	Alpine breed	Saanen breed
10	68.3	60.1

9	13.0	16.2

8	7.7	12.5

7	6.3	8.1

6	3.3	2.5

5	1.0	0.5

4	0.3	0.1

3	0.1	0

## Discussion and conclusion

The actual efficiency of the multi-step procedure will depend on the data set situation, *i.e.*, first, a comprehensive list of the NS males in activity and, second, complete pedigree recording for births from NS. Then, the inbreeding and coancestry coefficients used in the procedure will be underestimated on average. Because the AI organization that provided the data set is eager to conserve genetic variability during the selection process, its next objective should clearly be the improvement of pedigree recording. This demanding step might be initiated, provided that the present procedure is widely accepted and implemented by goat breeders. Meanwhile, it would be worthwhile to test and adapt to goat breeding the methods currently used for correcting inbreeding and coancestry coefficients for incomplete pedigrees [14,15], that do not consider the herd structure induced by natural service. The situation of incomplete pedigrees, if maintained in the future, will also make it difficult to monitor the true inbreeding and the true effect of the optimized scheme. This statement can be illustrated by turning back to the past [16,17]. The increase of females' inbreeding between 1994 and 2006 was very slow: from 1.50% to 1.34% in Saanen, from 1.26 to 1.36% in Alpine. This is hard to reconcile with the substantially faster increase of AI males' inbreeding: from 1.87 to 2.87% in Saanen and from 0.93 to 2.40% in Alpine. This difference might be due to the fact that pedigrees of AI males are generally more complete.

The situation of incomplete pedigrees and the need to provide recommendations accepted by breeders, with its multiple consequences, were the reasons why the authors departed from the general recommendation that selection schemes should be optimized for genetic gain at a given coancestry rate (usually between 0.5 and 1% per generation): see review in [18].

The selection schemes described in the literature for sustainable management of selected populations are generally simple [1,2,18], because this literature basically tends to clarify the general guidelines to be followed. They can be implemented with only minor complications in species such as poultry or fish [19], where selection and mating decisions can be centralized. In contrast, the scheme described here is dictated by goat biology and the farming system, long to describe and complex (5 distinct steps). The underlying principle was to propose a solution whenever breeders would face a decision involving genetics. This kind of situation was already encountered in pig breeding where AI and NS also coexist [4]. The only simplification that can be envisaged in the future is direct use of young bucks for AI without progeny-testing, based on the potential of genomic selection, following the dairy cattle example [20]. Consequently, a large number of optimization algorithms, all based on the simulated annealing principles, had to be constructed. Although each of them was fairly simple, their number led to a substantial programming load, a situation already met when optimizing diffusion of bull semen in the context of multi-trait selection [5].

Quite obviously, the successive simulated annealing procedures met the general objective, which was to promote a substantial genetic gain while containing development of inbreeding and coancestry. In some parts of the population, the latter coefficients were even reduced (see for instance the inbreeding coefficients of the programmed daughters in comparison with those of their dams). However, this effect will be transitory because, unavoidably, inbreeding and coancestry will increase again. Programming of inseminations for progeny-testing was also satisfactory because a future excellent connexion between flocks for the evaluation could be predicted. Due to these characteristics, implementation of the whole approach will start in the field in 2010. The adaptation of the procedure to genomic selection (replacing progeny-testing) will be straightforward and the resulting procedure will be much simplified. In any case, a better pedigree registration and a continuous monitoring of results in the field will be needed, especially concerning the asymptotic trend of inbreeding and coancestry (impossible to assess right now based on the present results). If unfavorable, the constraints used in the internal procedures should be modified.

## List of abbreviations

AI: Artificial Insemination; EBV: Estimated Breeding Value; NS: Natural Service; NSR: Replacement of natural service bucks.

## Authors' contributions

JJC defined and programmed the optimisation method. VC extracted the national files required (pedigrees and EBV). PM provided the data set and defined the desired operational constraints. IP contributed to the analysis and interpretation of data (test of different solutions for acceptability) and participated in writing the manuscript. All authors read and approved the final manuscript.
